# Genetic diversity of *Plasmodium falciparum* parasite by microsatellite markers after scale-up of insecticide-treated bed nets in western Kenya

**DOI:** 10.1186/s12936-015-1003-x

**Published:** 2015-12-09

**Authors:** Wangeci Gatei, John E. Gimnig, William Hawley, Feiko ter Kuile, Christopher Odero, Nnaemeka C. Iriemenam, Monica P. Shah, Penelope Phillips Howard, Yusuf O. Omosun, Dianne J. Terlouw, Bernard Nahlen, Laurence Slutsker, Mary J. Hamel, Simon Kariuki, Edward Walker, Ya Ping Shi

**Affiliations:** Division of Parasitic Diseases and Malaria, Center for Global Health, Centers for Disease Control and Prevention, Atlanta, GA USA; Liverpool School of Tropical Medicine, Liverpool, UK; Centre for Global Health Research, Kenya Medical Research Institute, Kisumu, Kenya; Atlanta Research and Education Foundation, Decatur, GA USA; Malawi-Liverpool-Wellcome Trust Clinical Research Programme, Blantyre, Malawi; President’s Malaria Initiative, Washington DC, USA; Michigan State University, East Lansing, USA

**Keywords:** *Plasmodium falciparum*, Population structure, Genetic diversity, ITNs, Transmission

## Abstract

**Background:**

An initial study of genetic diversity of *Plasmodium falciparum* in Asembo, western Kenya showed that the parasite maintained overall genetic stability 5 years after insecticide-treated bed net (ITN) introduction in 1997. This study investigates further the genetic diversity of *P. falciparum* 10 years after initial ITN introduction in the same study area and compares this with two other neighbouring areas, where ITNs were introduced in 1998 (Gem) and 2004 (Karemo).

**Methods:**

From a cross-sectional survey conducted in 2007, 235 smear-positive blood samples collected from children ≤15-year-old in the original study area and two comparison areas were genotyped employing eight neutral microsatellites. Differences in multiple infections, allele frequency, parasite genetic diversity and parasite population structure between the three areas were assessed. Further, molecular data reported previously (1996 and 2001) were compared to the 2007 results in the original study area Asembo.

**Results:**

Overall proportion of multiple infections (M_A_) declined with time in the original study area Asembo (from 95.9 %-2001 to 87.7 %-2007). In the neighbouring areas, M_A_ was lower in the site where ITNs were introduced in 1998 (Gem 83.7 %) compared to where they were introduced in 2004 (Karemo 96.7 %) in 2007. Overall mean allele count (M_AC_ ~ 2.65) and overall unbiased heterozygosity (*H*_*e*_ ~ 0.77) remained unchanged in 1996, 2001 and 2007 in Asembo and was the same level across the two neighbouring areas in 2007. Overall parasite population differentiation remained low over time and in the three areas at F_ST_ < 0.04. Both pairwise and multilocus linkage disequilibrium showed limited to no significant association between alleles in Asembo (1996, 2001 and 2007) and between three areas.

**Conclusions:**

This study showed the *P. falciparum* high genetic diversity and parasite population resilience on samples collected 10 years apart and in different areas in western Kenya. The results highlight the need for long-term molecular monitoring after implementation and use of combined and intensive prevention and intervention measures in the region.

**Electronic supplementary material:**

The online version of this article (doi:10.1186/s12936-015-1003-x) contains supplementary material, which is available to authorized users.

## Background

Insecticide-treated bed nets (ITNs), including long-lasting insecticide-treated bed nets (LLINs), are an important tool for malaria control [1]. In western Kenya, the efficacy of ITNs in reducing morbidity and all-cause mortality in children under 5 years of age was demonstrated previously [2–5]. Thereafter, a nationwide scale-up campaign to distribute ITNs in all 46 districts where malaria is endemic was undertaken [6]. By 2008, the Demographic Household Survey (DHS) showed overall 61 % Kenyan households owned at least one net of any kind and 47 % of children under 5-year-old slept under ITNs [7].

ITNs reduce malaria morbidity by killing or deterring mosquito vectors, thereby reducing the number of infective bites on human hosts [8]. To be optimally effective, ITNs require consistent and appropriate use and high community coverage in all age groups [2, 9]. Changes in malaria transmission due to the use of ITNs ultimately impact vector and parasite populations, but the effects, especially after scale-up of ITNs, on genetic diversity and parasite populations are still unclear [10, 11].

A previous study on the effects of transmission reduction by ITNs on parasite population structure using neutral MS markers in western Kenya, showed that *Plasmodium falciparum* maintained overall high genetic diversity and stability after 5 years of high ITN use [11] even in periods with substantial reduction in malaria transmission and decline of *Anopheles gambiae* [12–14]. Clinical and immunological aspects in the hosts coupled with factors such as changes in vector ecology and gene flow in vector and host migration, have been considered as potential factors affecting the parasite genetic stability [4, 11, 15, 16]. Seasonal change or geographical isolation that influence transmission may also affect *P. falciparum* genetic diversity and population structure [17–19].

Previous studies on genetic diversity over space and time conducted by others in Kenya reported limited time or geographical area effects on gene allelic frequencies of *P. falciparum* in western Kenya [20]. Although reasons for this occurrence are not clear, large local population sizes of *P. falciparum* with numerous reproductive units have been shown to contribute to extensive heterogeneity of the parasite with correspondingly limited or no genetic differentiation across different regions in high transmission areas in Africa [21–24]. However, other studies have shown that *P. falciparum* maintains a clonal structure with significant linkage disequilibrium (LD) in some high-transmission areas, indicating there are other factors influencing genetic diversity and population structure [25, 26]. Therefore, a long-term follow-up study of parasite genetic diversity and population structure in the same area and between adjacent geographic areas where ITNs were deployed more recently can help to understand the impact of transmission reduction following the scale-up of vector control programmes on parasite population.

The initial assessment of effects of ITNs on parasite genetic diversity on samples collected in 1996 and 2001 in the original study area, Asembo (ITN introduction in 1997) in Rarieda sub-county, Siaya county of western Kenya, showed *P. falciparum* maintained overall high genetic diversity but with locus-specific variation, which contributed to differences in population sub-structure [11]. The current study investigates further the genetic diversity of *P. falciparum* in samples from 2007 in Asembo and the data were compared to that from two neighbouring areas where ITNs were first introduced in 1998 (Gem, Gem sub-county) and 2004 (Karemo, Alego sub-county), respectively. Assessing differences in parasite genetic diversity between Asembo, Gem and Karemo would inform on different area effects based on different ITN coverage and/or usage, and the possible role of migration of parasites between the areas. Further, comparison of parasite diversity within Asembo in the 1996, 2001 and 2007 surveys would show any possible temporal effects of ITN application on parasite population in the same locality with decline in entomology inoculation rate (EIR) and malaria prevalence. The same eight single copy neutral microsatellite (MS) markers used previously were employed in this study [11] to assess the genetic diversity and population structure of *P. falciparum*. Assessments of changes between time points and between areas on *P. falciparum* population were quantified based on multiplicity of infection, allele frequency, unbiased heterozygosity, linkage disequilibrium (LD) and genetic differentiation.

## Methods

### Study areas and study samples

This was a follow-up study in Siaya county of western Kenya where a two-phase, community-based, ITN trial was conducted from 1996 to 2001 [3–5, 11]. The initial trial design and ITN introduction in the original area of Asembo in 1997 (Rarieda sub-county) and the second area of Gem in 1998 (Gem sub-county) has been described in detail previously [27]. In 2004, ITNs were implemented in the third area Karemo (Alego sub-county). During and after the ITN trial, annual malaria infection, cross-sectional surveys were conducted around the same times of the year to coincide with the rainy seasons [2, 4, 28].

**Fig. 1:**
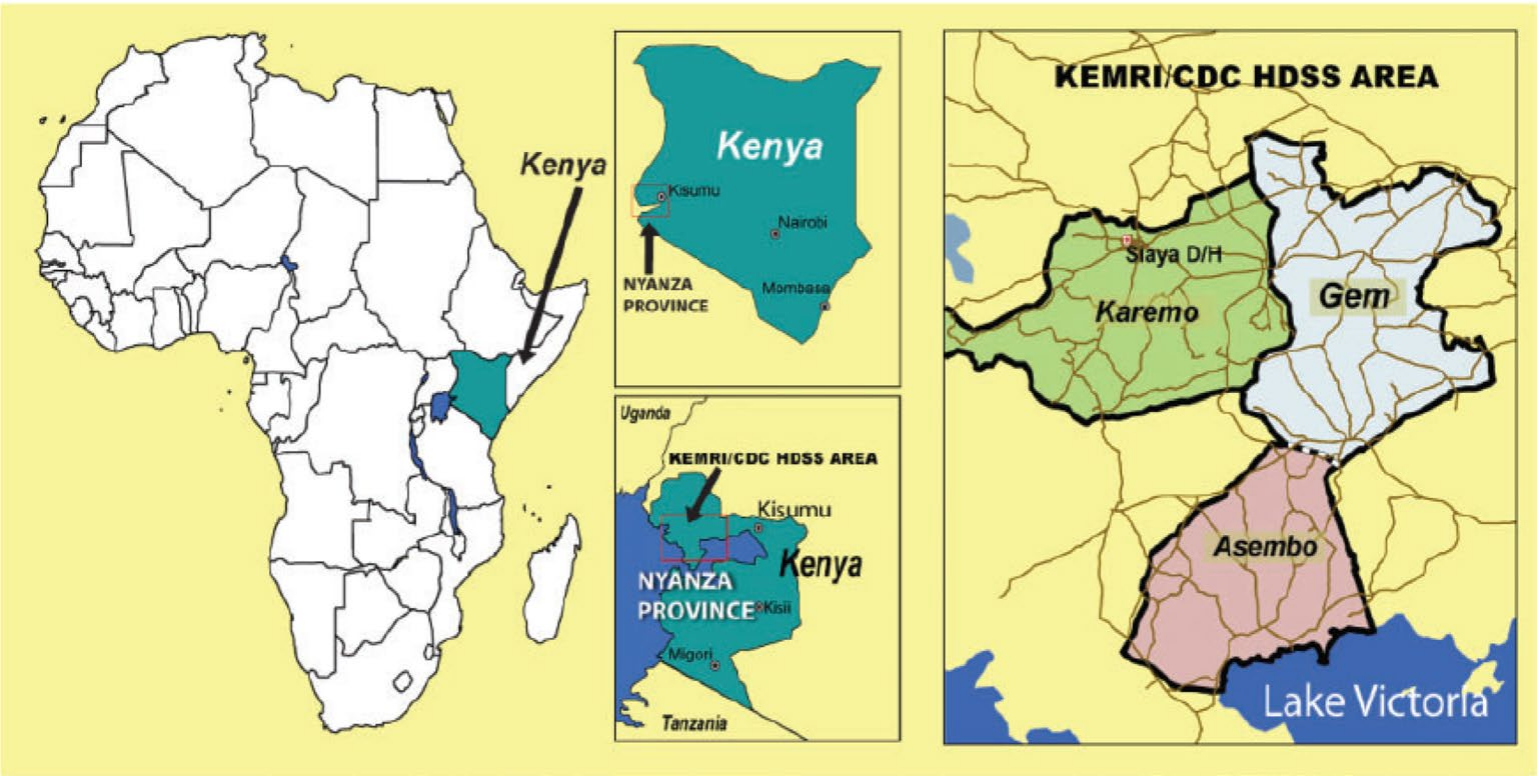
Geographic map showing the three study areas of Asembo, Gem and Karemo in Siaya County of western Kenya where insecticide-treated nets were introduced in 1997, 1998 and 2004, respectively

This study examines parasite diversity in samples collected from a cross-sectional survey conducted in 2007 in the Asembo and compares the results with those from Gem and Karemo areas. The geographic relationship of the three areas is shown in Fig. 1. Further, parasite diversity within Asembo in 1996, 2001 and 2007 was compared. In Asembo, after initial introduction of ITNs in 1997 the households with at least one ITN reached >95 % by 1999, and remained high through to 2008 [12]. However, while coverage was high, ITN usage was low among residents in the three study areas but the levels differed for each area. In the 2007 survey, the proportion of smear-positive participants reporting to have slept under any type of bed net (treated or untreated) the night prior to survey was 51 (Asembo), 44 (Gem) and 20 % (Karemo) while actual ITN usage was 49, 31 and 7 %, respectively [10]. In addition, following the initial introduction of ITNs in Asembo in 1997 and Gem in 1998, malaria transmission was reduced by 90 % at the early stages of ITNs trial, with the EIR falling from 61.3 infective bites per person per year to 1.3 in 2001 [4, 13]. In the 2007 survey, the EIR was estimated to be four in Asembo and Gem and 20 in Karemo (KEMRI/CDC, unpublished data). Prevalence of parasitaemia in children ≤5-year-old in Asembo was 70 and 34 % in 1996 and 2001, respectively [4, 5]. In the 2007 survey, parasitaemia prevalence in children <15-year-old was 35.8 % in Asembo, 45.4 % in Gem and 50.3 % in Karemo (KEMRI/CDC, unpublished data).

From the 2007 cross-sectional survey, a total of 235 smear-positive samples collected from children ≤15-year-old from Asembo (n = 56), Gem (n = 87) and Karemo (n = 92) were used for genetic analysis of parasites. For Asembo, the molecular data from 69 and 74 smear-positive samples collected in 1996 and 2001, respectively, from children ≤5-year-old and reported earlier were also included for further temporal comparison [11]. Dried blood spot (DBS) samples were collected on filter paper and stored at −80 °C. Parasite genomic DNA was extracted from one blood spot for each sample using the QIAmp DNA Mini kit (Qiagen, CA, USA) as per manufacturer’s instructions. Genomic DNA was stored at −20 °C until use.

The study was approved by the Ethical Review Committee of Kenya Medical Research Institute, Nairobi, Kenya, the Institutional Review Board of Michigan State University, East Lansing, MI, USA and the Institutional Review Board of CDC, Atlanta, GA, USA.

### Microsatellite (MS) markers and genotyping

The genetic diversity of *P. falciparum* parasites was assessed by scoring eight single copy neutral MS loci located on different chromosomes for all the samples as reported previously [29]. The selected MS markers, the primer sequences and amplification conditions used in this study have been described previously [11, 29, 30]. Briefly, five neutral markers (Poly-α, PfPK2, ADL, TAA60 and TAA109), one MS marker linked to the protein expressed during the gametocyte maturation stages of *P. falciparum* (Pfg377) and two MS linked to genes of asexual stage antigens under possible natural immune selection (EBP and P195) were used. All MS scoring in base length and peak height, and quantification of multiple alleles used the same method as described previously [11]. Briefly, MS base pair length and peak height were quantified by GeneMapper software (ABI). For each locus, allele identity was obtained from all peaks above 200 fluorescent units (fu). The highest peak was identified as the predominant allele, while minor alleles were determined at peak heights of ≥30 % of the predominant allele meeting the 200 fu criteria. Amplification for the eight MS ranged from 90 to 100 % and samples failing to amplify for any of the MS was reported as missing and not used for haplotype definition.

### Parameters measured and data analysis

All microsatellite raw data were managed using the Excel Microsatellite Tool Kit [31] and consequently formatted for other genetic analyses software programs. For multiple infections, both the predominant and minor alleles were counted to quantify the proportion of infections with more than one allele (M_A_), while the highest number of allele count detected by any of the MS comprised the mean allele counts (M_AC_). Differences in both M_A_ and M_AC_ between time points or between areas were assessed using Pearson’s Chi square and one way analysis of variance (ANOVA). Conversely, only the predominant allele in each locus was used to analyse all other parameters of genetic diversity and population structure, including unbiased heterozygosity (*H*_*e*_) and allele richness calculated as the average number of alleles per locus, LD and genetic differentiation (F_ST_) [11]. Multiple comparisons were corrected using Bonferroni correction for all tests where applicable. Allele richness and allele frequency were obtained using FSTAT [32]. Unbiased heterozygosity (*H*_*e*_), sampling variance of *H*_*e*_, was calculated as described previously [33] with *p*-levels obtained from *z* absolute values from the standard error (SE) of sampling variance. The LD measures the degree of association between gene pairs or among gene loci (structured population when LD is significant) assuming a null hypothesis of no association in random genetic recombination (population admixture when LD is insignificant). Pairwise LD, measuring the degree of association between MS, was obtained using ARLEQUIN [34]. Multilocus LD, measuring non-random association among all loci, was assessed with the index of association ($I_{A}^{S}$) using LIAN program [35]. Multilocus LD tests the differences in variance of observed (V_d_) and the variance expected (V_e_) at LD, assuming a null hypothesis (H_o_) derived from 10,000 simulated data sets: V_d_ = V_e_. Genetic differentiation (F_ST_) was tested by the Fisher’s exact test using the GenePop Program [36]. The F_ST_ is a measure of the sum of genetic variability within and between parasite populations based on differences in allele frequencies. Categorization for F_ST_ was defined as no differentiation or low differentiation (F_ST_ < 0.05), moderate differentiation (≥0.05 F_ST_ < 0.15) and great differentiation (F_ST_ ≥ 0.15) as described previously and applied previously [11, 37].

## Results

Since the Asembo 2007 survey comprised children up to 15 years of age, initial data were stratified by age (≤5- and >5-year-old) and tested for differences in parasite genetic diversity (Additional file 1: Table S1, Additional file 2: Table S2). As no significant differences in parasite diversity were detected by age, the molecular data from the 2007 survey were combined and analysed as one population in comparison with 1996 and 2001 surveys. In addition, a previous study conducted by us reported no difference in multiple infections between 1996 and 2001 [11] and initial temporal analysis in this study showed no significant variations in *H*_*e*_, but there were some differences in LD and F_ST_ between parasite populations from the three time points. For brevity, therefore, temporal data presented below focused on comparison of the parameters of multiple infection and genetic diversity only between 2001 and 2007 while comparison of LD and F_ST_ of parasite populations among 1996, 2001 and 2007 surveys are presented.

### Multiple infections

Overall proportion of infections with more than one allele (M_A_) by any of the eight MS in the three study areas was over 80 %, a reflection of a highly polyclonal *P. falciparum* parasite population. In the different area analyses, the overall M_A_ was significantly higher in Karemo at 96.7 % compared to Asembo (87.7 %) and Gem (83.7 %) (*p* = 0.01). In contrast, the overall mean allele counts (M_AC_) were similar (*p* = 0.53) at 2.76 (Asembo), 2.55 (Gem) and 2.68 (Karemo). For individual MS, only P195 showed significantly higher M_A_ and M_AC_ in Karemo compared to both Asembo and Gem (*p* = 0.01). This pattern was reversed for both Pfg377 and PfPK2 where both M_A_ and M_AC_ were significantly lower in Karemo compared to both Asembo and Gem as shown in Table 1a. No differences were detected for all other loci for the same measures in the three study areas.

**Table 1:** Comparison of proportion of multiple alleles (M_A_) and mean allele counts (M_AC_) of parasite populations in (a) Asembo, Gem and Karemo areas, 2007 survey and (b) Asembo area in 2001 and 2007 surveys

(a) Asembo (n = 57), Gem (n = 87), Karemo (n = 92)				*p* value <0.05	
Locus	Area	% M_A_	M_AC_ ± SE	% M_A_*	M_AC_*
Poly-α	Asembo	59.6 %	2.22 ± 0.17	0.92	0.28
	Gem	57.0 %	2.08 ± 0.13		
	Karemo	59.8 %	1.92 ± 0.10		
Pfg377	Asembo	37.5 %	1.45 ± 0.09	0.06	*0.02*
	Gem	32.9 %	1.40 ± 0.06		
	Karemo	20.7 %	1.24 ± 0.05		
PfPK2	Asembo	53.6 %	1.96 ± 0.15	*0.01*	*0.01*
	Gem	45.8 %	1.87 ± 0.12		
	Karemo	28.1 %	1.33 ± 0.06		
ADL	Asembo	46.9 %	1.70 ± 0.12	0.79	0.13
	Gem	53.9 %	2.01 ± 0.15		
	Karemo	50.0 %	1.71 ± 0.09		
EBP	Asembo	41.2 %	1.49 ± 0.08	0.50	0.76
	Gem	31.3 %	1.41 ± 0.07		
	Karemo	36.8 %	1.45 ± 0.07		
P195	Asembo	29.6 %	1.37 ± 0.08	*0.00*	*0.01*
	Gem	22.2 %	1.27 ± 0.05		
	Karemo	51.7 %	1.58 ± 0.06		
TAA60	Asembo	52.6 %	1.84 ± 0.13	0.75	0.41
	Gem	45.9 %	1.69 ± 0.09		
	Karemo	46.0 %	1.64 ± 0.09		
TAA109	Asembo	44.4 %	1.53 ± 0.08	0.50	0.64
	Gem	35.8 %	1.62 ± 0.11		
	Karemo	44.0 %	1.68 ± 0.11		
Overall	Asembo	87.7 %^a^	2.76 ± 0.16^b^	*0.01*	0.53
	Gem	83.7 %^a^	2.55 ± 0.14^b^		
	Karemo	96.7 %^a^	2.68 ± 0.08^b^		

(b) Locus	% M_A_		M_AC_ ± SE		*p* values	
	Asembo 2001	Asembo 2007	Asembo 2001	Asembo 2007	% M_A_*	M_AC_*
Poly-α	71.6 %	59.6 %	2.40 ± 0.14	2.22 ± 0.17	0.11	0.19
Pfg377	52.8 %	37.5 %	1.67 ± 0.09	1.45 ± 0.09	0.06	0.08
PfPK2	47.3 %	53.6 %	1.74 ± 0.11	1.96 ± 0.15	0.30	0.37
ADL	50.0 %	46.9 %	1.54 ± 0.07	1.70 ± 0.12	0.44	0.58
EBP	61.1 %	41.2 %	2.10 ± 0.14	1.49 ± 0.08	*0.02*	*0.01*
P195	47.9 %	29.6 %	1.62 ± 0.09	1.37 ± 0.08	*0.03*	*0.02*
TAA60	59.5 %	52.6 %	1.96 ± 0.12	1.84 ± 0.13	0.27	0.24
TAA109	76.7 %	44.4 %	2.26 ± 0.12	1.53 ± 0.08	*0.01*	*0.01*
Overall	95.9 %^a^	87.7 %^a^	3.1 ± 0.12^b^	2.76 ± 0.16^b^	*0.03*	0.16

Asembo, Gem and Karemo denotes years after introduction of ITNs; 10, 9 and 3 years, respectively

Asembo 2001 (n = 74); Asembo 2007 (n = 56)

% M_A_ is the proportion infections with more than one allele in each locus while the M_AC_ is the mean allele count with the respective standard error (SE) at each locus. The superscript a marks the overall proportion of infections with at least two alleles while the superscript b marks the overall mean of the highest number of allele count detected by any of the eight microsatellites.  % M_A_* and M_AC_* show the *p* values for the differences in the proportion of multiple alleles and mean allele counts between parasite populations from the three areas or between 2001 and 2007 surveys. Numbers highlighted in italics show significance levels <0.05

For the temporal effect analysis within Asembo, the overall M_A_ dropped from 95.9 % in 2001 to 87.7 % in 2007 (*p* = 0.03), but the reduction in the overall M_AC_ (from 3.1 in 2001 to 2.8 in 2007) was not statistically significant. At individual MS, there were significant decreases in M_A_ and M_AC_ from 2001 to 2007 at the P195 (47.5 %, 1.62–29.6 %, 1.37; *p* < 0.05), EBP (61.1 %, 2.10–41.2 % and 1.49; *p* < 0.05), and TAA109 (76.7 %, 2.26–44.4 % and 1.48; *p* < 0.05), respectively. No other significant changes, including Pfg377 MS, were observed between the two time periods as shown in Table 1b.

### Genetic diversity

Allele size and composition for the eight MS in parasite populations from each area in the 2007 survey is shown in Additional file 3: Figure S1. The number of alleles per locus based on allele size reflected the extensive and high genetic diversity in *P. falciparum* population in the three study areas. Allele numbers per locus ranged from a low of five for the Pfg377 locus in Asembo and Karemo to a high of 19 for the Poly-α locus in Karemo. The overall *H*_*e*_ was approximately 0.8 in the three study areas as shown in Table 2a. Within the individual MS, *H*_*e*_ was significantly lower at P195 locus in Gem (*H*_*e*_ = 0.65) than in both Asembo (*H*_*e*_ = 0.74) and Karemo (*H*_*e*_ = 0.74). No significant difference in *H*_*e*_ for other individual MS markers between the areas was detected (Table 2a). Similarly, no significant differences in overall and loci specific *H*_*e*_ were observed in the samples from Asembo area between the 2001 and 2007 time points (Table 2b).

**Table 2:** Genetic diversity of parasites in (a) Asembo, Gem and Karemo areas, 2007 survey and, (b) Asembo 2001 and 2007 surveys

(a) Locus	Asembo		Gem		Karemo		*p* value		
	Allele no. and (richness)	*H*_*e*_ (SE)	Allele no. and (richness)	*H*_*e*_ (SE)	Allele no. and (richness)	*H*_*e*_ (SE)	Asembo/Gem	Asembo/Karemo	Gem/Karemo
Polya	16 (15.94)	0.91 (0.0193)	17 (16.78)	0.89 (0.0279)	19 (18.85)	0.88 (0.0315)	0.620	0.469	0.814
Pfg377	5 (4.99)	0.47 (0.0726)	6 (5.74)	0.41 (0.0648)	5 (4.72)	0.32 (0.059)	0.472	0.073	0.316
PfPK2	10 (10)	0.85 (0.0243)	11 (10.44)	0.82 (0.0209)	12 (11.32)	0.84 (0.0221)	0.353	0.749	0.530
ADL	12 (12)	0.88 (0.0177)	14 (13.71)	0.90 (0.0114)	16 (15.28)	0.91 (0.0089)	0.205	0.075	0.550
EBP	8 (8)	0.82 (0.0562)	14 (13.4)	0.88 (0.0388)	11 (10.84)	0.83 (0.0232)	0.426	0.886	0.312
P195^a^	7 (6.99)	0.74 (0.0249)	6 (5.83)	0.65 (0.0206)	6 (5.99)	0.74 (0.0209)	*0.005*	0.957	*0.003*
TAA60	8 (7.98)	0.81 (0.0249)	9 (8.42)	0.79 (0.0206)	7 (6.96)	0.79 (0.0209)	0.561	0.550	0.983
TAA109	9 (8.97)	0.79 (0.0249)	12 (11.28)	0.83 (0.0169)	14 (13.17)	0.83 (0.0239)	0.124	0.178	0.993
Overall		0.78 (0.0495)		0.77 (0.0060)		0.77 (0.0661)	0.813	0.859	0.972

(b) Locus	Asembo-2001		Asembo-2007		*p* value
	Allele no. and (richness)	*H*_*e*_ (SE)	Allele no. and (richness)	*H*_*e*_ (SE)	
Poly-α	17 (16.92)	0.92 (0.0243)	16 (15.94)	0.91 (0.0193)	0.653
Pfg377	3 (3.00)	0.57 (0.0984)	5 (4.96)	0.47 (0.0726)	0.489
PfPK2	12 (11.92)	0.83 (0.0497)	10 (10.00)	0.85 (0.0243)	0.752
ADL	14 (13.96)	0.89 (0.0191)	12 (12.00)	0.88 (0.0177)	0.623
EBP	6 (5.99)	0.82 (0.0193)	8 (8.00)	0.82 (0.0562)	0.976
P195	11 (10.91)	0.75 (0.0241)	7 (6.99)	0.74 (0.0249)	0.733
TAA60	17 (16.87)	0.85 (0.0322)	8 (7.98)	0.81 (0.0249)	0.344
TAA109	11 (10.94)	0.77 (0.0372)	9 (8.97)	0.79 (0.0249)	0.691
Overall		0.79 (0.0380)		0.78 (0.0495)	0.625

Asembo, Gem and Karemo denotes years after introduction of ITNs; 10, 9 and 3 years, respectively

Comparison of genetic diversity between areas and between years was based on the number of alleles, allele richness (between areas only), unbiasied heterozygosity (*H*_*e*_) and standard error (SE) [31]. Standard error was calculated to generate a *p* value for statistical testing of differences in *H*_*e*_

*p* value <0.05 are in italics

^a^Denotes locus with significantly different *H*_*e*_ between areas

### Pairwise and multilocus LD

Overall, results of 28 pairwise comparisons for each area showed that LD was significant (*p* ≤ 0.0018) for 16, 14 and 15 MS pairs in Asembo, Gem and Karemo, respectively. Of note, Pfg377, the MS flanking the gene relating to gametocyte maturation, had the least number of significant pairwise LD (only paired with P195 and ADL; *p* ≤ 0.0018) in Gem and Karemo, respectively, while no significant LD was observed in Asembo. Conversely, the remaining MS had at least ten of the significant pairwise LD in all three areas as shown in Additional file 4: Table S3.

Within Asembo, the 16 MS pairs showed significant LD in the 2007 survey while only six MS pairs had significant LD in the 2001 survey. The high number of significant pairwise LD in 2007 was similar to that observed in the baseline survey (1996) where 14 pairs of MS showed significant LD. However, despite these overall changes in the number of significant pairwise LD in the different time points, LD at Pfg377 locus again showed the least number with only four significant pairs in the three time points [P195 and ADL in 1996, P195 and EBP in the 2001 and no pairs in 2007 survey (*p* ≤ 0.0018; Additional file 4: Table S3 and Additional file 5: Table S4)]. This suggests possible consistent locus specific diversity at the Pfg377, which shows higher random association with other MS alleles and therefore less LD.

Multilocus LD, testing non-random association on all loci, among the three study areas showed diverse results. In Asembo, the variance in observed (V_d_) of 1.268 was significantly higher than the variance expected (V_e_) which was 1.133 (*p* = 0.03) with an index of association ($I_{A}^{S}$) of 0.017, suggesting a significant multilocus LD. In contrast, multilocus LD was not significant in either Gem or Karemo where $I_{A}^{S}$ was −0.003 and 0.001, respectively (Table 3a). The results suggest a more structured *P. falciparum* population in Asembo while parasite population in Gem and Karemo show more admixtures in 2007.

**Table 3:** Estimates of multilocus LD for *P. falciparum* populations in (a) Asembo, Gem and Karemo areas, 2007 survey and, (b) Asembo 1996, 2001 and 2007 surveys

(a) Test factor	Areas		
	Asembo	Gem	Karemo
V_D_	1.268	1.1278	1.104
V_e_	1.133	1.1502	1.099
$I_{A}^{S}$	0.017	−0.003	0.001
Testing (H_0_: V_d_ = V_e_)			
Var (V_D_)	0.003	0.002	0.002
*p* value	0.030	0.680	0.440

(b) Test factor	Survey year		
	Asembo-1996	Asembo-2001	Asembo-2007
VD	1.209	1.183	1.268
Ve	1.087	1.180	1.133
$I_{A}^{S}$	0.016	0.001	0.017
Testing (H_0_: V_d_ = V_e_)			
Var (VD)	0.002	0.002	0.003
*p value*	0.01	0.57	0.030

Multilocus LD for the eight MS markers for (a) Asembo, Gem and Karemo 2007 and for (b) Asembo 1996, 2001 and 2007 surveys. *p* values shown are derived from Monte Carlo simulations methods for $I_{A}^{S}$ showing departure from null hypothesis of no association (0) for each population

Within Asembo, the multilocus LD reflected the previous pattern observed in the pairwise LD. Multilocus LD was significant in 2007 survey ($I_{A}^{S}$ = 0.017) in contrast to the previous 2001 survey ($I_{A}^{S}$ = 0.001) but similar to the 1996 baseline survey ($I_{A}^{S}$ = 0.016) as shown in Table 3b. These results suggest that the *P. falciparum* population while structured in 1996, had more admixture in 2001, but was more structured 10 years after the introduction of ITN use in Asembo.

### Genetic differentiation

In assessing different area effects, the overall genetic differentiation within Asembo, Gem and Karemo was low (F_ST_ = 0.021). When individual MS were analysed, only P195 MS showed moderate genetic differentiation (F_ST_ ≥ 0.05 < 0.15) between any two areas in 2007. All other individual MS showed low differentiation (F_ST_ < 0.05) that was not significant between the three study areas (Table 4a). A number of the F_ST_ negative values were observed in the comparisons between the three areas. This indicated different parasite populations being closer to each other between than within areas.

**Table 4:** Genetic differentiation index (F_ST_) for *P. falciparum* populations (a) in Asembo, Gem and Karemo areas, 2007 survey and, (b) in Asembo between 1996 and 2007, and 2001 and 2007 surveys

(a) Locus	Area1	Area2	F_ST_	Levels of differentiation
Poly-α	Asembo	Gem	−0.003	Low
		Karemo	0.001	Low
	Gem	Karemo	−0.004	Low
Pfg377	Asembo	Gem	−0.003	Low
		Karemo	0.005	Low
	Gem	Karemo	−0.002	Low
PfPK2	Asembo	Gem	0.007	Low
		Karemo	0.001	Low
	Gem	Karemo	0.009	Low
ADL	Asembo	Gem	0.003	Low
		Karemo	0.001	Low
	Gem	Karemo	0.006	Low
EBP	Asembo	Gem	0.018	Low
		Karemo	0.003	Low
	Gem	Karemo	0.002	Low
P195	Asembo	Gem	**0.059**	Moderate
		Karemo	**0.100**	Moderate
	Gem	Karemo	**0.133**	Moderate
TAA60	Asembo	Gem	−0.006	Low
		Karemo	−0.011	Low
	Gem	Karemo	−0.009	Low
TAA109	Asembo	Gem	0.032	Low
		Karemo	0.022	Low
	Gem	Karemo	−0.002	Low
ALL	Asembo		0.021	Low
	Gem		0.021	Low
	Karemo		0.021	Low

(b) Locus	Survey year		*F*_ST_	Levels of differentiation
Poly-α	1996	2007	0.005	Low
	2001	2007	−0.006	Low
Pfg377	1996	2007	0.022	Low
	2001	2007	0.022	Low
PfPK2	1996	2007	0.009	Low
	2001	2007	−0.001	Low
ADL	1996	2007	0.006	Low
	2001	2007	0.009	Low
EBP	1996	2007	0.007	Low
	2001	2007	0.013	Low
P195	1996	2007	**0.105**	Moderate
	2001	2007	**0.141**	Moderate
TAA60	1996	2007	0.037	Low
	2001	2007	**0.057**	Moderate
TAA109	1996	2007	0.037	Low
	2001	2007	0.028	Low
Overall	1996	2007	0.026	Low
	2001	2007	0.040	Low

Genetic differentiation index (F_ST_) was assessed at each MS between (a) any two areas of Asembo, Gem and Karemo and (b) in Asembo between 1996, 2001 and 2007 surveys’ parasite populations. This was based on the null hypothesis that alleles are drawn from the same distribution in any of the parasite populations tested. The levels were defined as little-to-low F_ST_ (<0.05), moderate (≥0.05 to <0.15) and great differentiation (≥0.15) as described previously [37]

Moderate F_ST_ was highlighted in bold

For assessing the temporal effects on genetic differentiation, the overall genetic differentiation was relatively higher in the 2001 and 2007 time points (F_ST_ = 0.040) compared to the 1996/2007 testing where F_ST_ was 0.026. Incidentally, as reported previously, F_ST_ was also low in the 1996 and 2001 testing at F_ST_ 0.027 [11]. The overall F_ST_ results show that over the three time points spanning 10 years there was only limited differences in allele frequencies resulting in insignificant parasite population differentiation in Asembo area. At the individual MS, P195 locus, as in the different areas analyses, showed consistently moderate differentiation for the paired time point comparisons between the years 1996, 2001 and 2007 (Table 4b). Although differentiation at this locus could have contributed to the differences in overall F_ST_ in Asembo, the effect of a single locus in the overall population differentiation remained low considering the 10-year period since introduction of ITNs.

## Discussion

This study was aimed at assessing changes on *P. falciparum* population genetic diversity after scale-up of ITNs in three adjacent geographic areas: Asembo, Gem and Karemo, where ITNs were introduced at different times: Asembo in 1997, Gem in 1998 and Karemo in 2004. The study further examined temporal changes on parasite diversity within Asembo. Overall proportion of multiple infections (M_A_) dropped from 95.9 % in 2001 to 87.7 % in 2007. The M_A_ levels were similar in Asembo (87.7 %) and Gem (83.7 %) but significantly higher in Karemo (96.7 %) in 2007. However, the overall mean allele count M_AC_ remained unchanged at around 2.65 in the three areas and at the different time points. Further, after 10 years of sustained ITNs use (1997–2007), the genetic diversity measured by *H*_*e*_ remained unchanged at approximately the same level over time and in the three areas (*H*_*e*_ ~ 0.78). Additionally, there was low parasite population differentiation for the three areas (F_ST_ = 0.021) and over time (F_ST_ < 0.04). The only slight difference observed was that in Asembo there was less significant pairwise LD and insignificant multilocus LD in 2001 compared to 1996 (baseline) and 10 years later (2007).

Initial hypothesis of this study was that malaria transmission reduction, mainly by ITNs, would decrease parasite diversity. However, in spite of relative differences in duration of ITN implementation, use of ITNs and EIR between Karemo and Asembo (also Gem), the overall *H*_*e*_ observed in the three study areas remained high. The similarly high *H*_*e*_ for *P. falciparum* using neutral MS markers was reported in Kombewa and other areas of western Kenya, including Kapsulu, Kodera, Rangwe, Ringa, and Rota villages in surrounding counties, although no data on ITN usage were presented [20, 38]. The high *H*_*e*_, coupled with low overall genetic differentiation between areas in this study suggest the possible existence of vibrant reproductive units that maintain the high diversity within *Plasmodium* parasite pools. The high diversity and limited genetic differentiation also suggest gene flow is likely to be a major factor in maintaining vast parasite pools in the geographic region. The negative F_ST_ results observed in this study further illustrate the extent of admixture and cross-breeding within parasite populations in the three areas. Gene flow due to human migration was reported previously as a contributing factor to a resilient *Plasmodium* parasite population in western Kenya [39]. Demographic data also confirm steady migration in the study areas, with an average of 130 per 1000 person years out-migrating, and 20 per 1000 person years in-migrating annually [40]. In addition, sub-microscopic infection and gametocyte reservoirs could indirectly contribute to genetically diverse, yet stable, parasite population observed here in the three study areas. Microscopically detectable parasitaemia, including both asexual and sexual stage parasites, could significantly underestimate the true level of parasite transmission. For example, with scale-up of malaria controls in western Kenya, the proportion of sub-microscopic infections at community level remains high and sub-microscopic gametocyte carriers are substantial in both Asembo and Karemo areas (Zhou et al., in prep) that could serve as potential transmission reservoirs, consequently maintaining parasite diversity. Indeed, a model on transmission dynamics of *P. falciparum* from hosts with a large pool of sub-microscopic asexual parasites and gametocytes predicted high uninterrupted transmission even with scaled-up LLIN coverage [41]. This robust but obscure transmission, coupled with possible over-representation of stable parasite reproductive units and gene flow due to geographical proximity of the study areas, may explain the overall genetic stability in the three study areas.

Within Asembo the overall *He* remained high and stable in 1996, 2001 [11] and 2007 surveys in which period malaria prevalence declined from 70 to 36 % and EIR from 61 to 4. There was also no difference in M_AC_ and the overall level of population differentiation (F_ST_) remained low over the three time points. A notable change was observed only in 2001 in the LD parameters with less significant pairwise LD and insignificant multilocus LD compared to 1996 (baseline) and 10 years later (2007). It is possible that sudden changes in parasite population due to the initial transmission reduction by introduction of ITNs in 1997 could allow minor populations with different allele frequencies to become dominant which might result in admixture parasite population (insignificant LD) in 2001. It is also likely that sulfadoxine-pyrimethamine (SP) and chloroquine (CQ) resistance contributed to malaria transmission [42], resulting in sustainability of parasite diversity although malaria prevalence measured by microscopy declined over the time. A study conducted in Papua New Guinea showed a strong association between multiplicity of infections and genetic diversity which was not related to prevalence, and the genetic diversity was maintained at high levels with no visible seasonal variation [43]. Other studies show that scaled-up malaria control and reduced transmission result in focal clusters of high transmission, which act as consistent parasite reservoirs [42]. Taken together, this suggests lack of direct correlation between declining prevalence (or EIR) and decreased genetic diversity [44]. Therefore, molecular monitoring is critical especially where prevalence as measured by microscopy has reduced significantly yet sub-microscopic infection that contributes malaria transmission continues [45].

While overall *H*_*e*_ was similar in the three areas and stable over time, there were differences in overall M_A_. In Karemo, where ITNs were introduced since 2004 (the shortest time) and with the lowest use at 20 %, M_A_ was significantly higher at 96.7 % than in Asembo at 87.7 % (ITN introduction in 1997 with 51 % use), or Gem 83.7 % with ITN introduction in 1998 and 44 % use. Similarly, where temporal effects were assessed in Asembo, M_A_ also significantly decreased from 2001 (95.9 %) to 2007 (87.7 %) after 10 years since introduction of ITNs. However, the temporal and area differences in M_A_ were not substantial to affect the overall *H*_*e*_, suggesting that multiple infections could be confounded by within-host competition among the parasite clones that are under selection by drug pressure, host immune pressure or parasites in different species of mosquito vector, all of which would be influenced by various malaria control measures. The results also suggest that measuring multiple infections could serve as an early indicator for change of malaria transmission.

In this study, there were also a few significant differences by different measures for individual MS markers, P195, Pfg377, EBP, PfPK2 and TAA109, among the three areas and/or between time points. Although the reasons for variations in EBP, PfPK2 and TAA109 loci between time points and among the areas are unclear, it is notable that P195 locus, the MS flanking gene encoding for asexual stage antigen under possible immune selection [46], consistently showed significant differences by different measures, suggesting that the P195 could be robust in reflecting alteration of parasite population due to subtle differences in the host’s immunity influenced by malaria exposure. In addition, Pfg377 MS locus, another important marker linked to a protein gene exclusively expressed during maturation of gametocytes [47], showed the least number of significant pairwise LD in three survey time points and across three areas. The results suggest a high random association of Pfg377 with other MS alleles to adjust gametocyte-related diversity as the parasites adapt to changes in transmission, which further indicates this marker could potentially be used as an adaptive marker for measuring change in transmission in future.

This study has a few limitations. The effect on parasite diversity was extrapolated based on neutral MS markers that may not fully capture the dynamism of the parasite population in the face of different control measures that include ITN use and drug pressure, which would further shape host immunity. Geographic proximity of the three study areas could have limited the ability to detect significant area divergences in the parasite populations. This is a cross sectional survey and while ITN coverage was high the actual use in the nights before sampling showed low usage (all below 50 %) which limited dissecting the impact of ITNs on parasite diversity. The temporal comparison was also limited as only one area had at least 10 years of ITN use. Further studies on parasite genetic diversity/structure for longer periods and in wider geographical regions, as well as use of other unique and robust genetic markers of parasites [48, 49], will be necessary to understand transmission dynamics and other factors that continue to sustain the high parasite diversity despite the use of ITNs/LLINs and case management by drug therapy in western Kenya.

## Conclusion

This study has shown the overall high genetic diversity and stability of *P. falciparum* over 10 years and across three different areas after scale-up of ITNs. The parasite resilience was reflected by a change in LD in Asembo at mid-point (5 years) but not at the 10-year time point. In addition to the gene flow between areas, other possible factors that might be attributed to the high and stable diversity of parasite population mainly are sub-microscopic infection and large gametocyte reservoir. Theoretically, a dramatic transmission reduction as a result of using multiple and intensive prevention and intervention measures can decrease parasite genetic diversity by creating a bottleneck effect on parasite population; for this to happen in western Kenya, such combined and intensive prevention and intervention measures must be sustained and cover wide geographic areas.

## Additional files

10.1186/s12936-015-1003-x Comparison of the frequency of multiple alleles in Asembo 1a), Gem 1b) and Karemo 1c) by age.
10.1186/s12936-015-1003-x Unbiased expected heterozygosity for three study areas by age.
10.1186/s12936-015-1003-x Allele sizes and compositions (base pairs, *X*-axis) and frequency distribution (*Y*-axis) of eight individual MS for *P. falciparum* parasite populations from Asembo, Gem and Karemo areas, 2007.
10.1186/s12936-015-1003-x Comparison of pairwise LD for *P. falciparum* populations in a) Aembo, Gem, and Karemo Areas, 2007 survey and, b) Asembo 1996, 2001 and 2007 surveys.
10.1186/s12936-015-1003-x Number of gene copies and number of alleles per locus used in LD analyses for Asembo, Gem and Karemo.
