# Supplementary material for: Genetic diversity of Plasmodium falciparum parasite by microsatellite markers after scale-up of insecticide-treated bed nets in western Kenya
Source: Malar J. 2015 Dec 9;14:495. doi: 10.1186/s12936-015-1003-x (PMC4675068; doi:10.1186/s12936-015-1003-x)
Supplement: Supplementary file 1 — 10.1186/s12936-015-1003-x Comparison of the frequency of multiple alleles in Asembo 1a), Gem 1b) and Karemo 1c) by age. [file 12936_2015_1003_MOESM1_ESM.docx]

|  | | | | | |
| --- | --- | --- | --- | --- | --- |
|  |  |  | Multiple Alleles | | Total |
|  |  |  | 1 | 2 |  |
| Asembo | Under 5 years | Count | 4 | 17 | 21 |
|  |  | % within | 19.0% | 81.0% | 100.0% |
|  |  | % within Multiple Alleles | 44.4% | 37.8% | 38.9% |
|  |  | % of Total | 7.4% | 31.5% | 38.9% |
|  | Above 5 years | Count | 5 | 28 | 33 |
|  |  | % within | 15.2% | 84.8% | 100.0% |
|  |  | % within Multiple Alleles | 55.6% | 62.2% | 61.1% |
|  |  | % of Total | 9.3% | 51.9% | 61.1% |
| Total | | Count | 9 | 45 | 54 |
|  |  | % within | 16.7% | 83.3% | 100.0% |
|  |  | % within Multiple Alleles | 100.0% | 100.0% | 100.0% |
|  |  | % of Total | 16.7% | 83.3% | 100.0% |
|  | | *P* value | 0.723 | | |

Additional file 1: Table S1 Comparisons of the Frequency of Multiple Alleles in Asembo 1a), Gem 1b) and Karemo 1c) by Age

Table 1 a) Asembo, Multiple Alleles Cross-tabulation by Age

Table S1 b) Gem, Multiple Alleles Cross-tabulation by Age

|  | | | | | |
| --- | --- | --- | --- | --- | --- |
|  |  |  | Multiple Alleles | | Total |
|  |  |  | 1 | 2 |  |
| Gem | Under 5 years | Count | 5 | 31 | 36 |
|  |  | % within | 13.9% | 86.1% | 100.0% |
|  |  | % within Multiple Alleles | 38.5% | 43.1% | 42.4% |
|  |  | % of Total | 5.9% | 36.5% | 42.4% |
|  | Above 5 years | Count | 8 | 41 | 49 |
|  |  | % within | 16.3% | 83.7% | 100.0% |
|  |  | % within Multiple Alleles | 61.5% | 56.9% | 57.6% |
|  |  | % of Total | 9.4% | 48.2% | 57.6% |
| Total | | Count | 13 | 72 | 85 |
|  |  | % within | 15.3% | 84.7% | 100.0% |
|  |  | % within Multiple Alleles | 100.0% | 100.0% | 100.0% |
|  |  | % of Total | 15.3% | 84.7% | 100.0% |
|  | | *P* value | 1.000 | | |

Table S1 c) Karemo, Multiple Alleles Cross-tabulation by Age

|  |  |  | Multiple Alleles | | Total |
| --- | --- | --- | --- | --- | --- |
|  |  |  | 1 | 2 |  |
| Karemo | Under 5 years | Count | 0 | 37 | 37 |
|  |  | % within | .0% | 100.0% | 100.0% |
|  |  |  |  |  |  |
|  |  | % within Multiple Alleles | .0% | 43.0% | 41.6% |
|  |  | % of Total | .0% | 41.6% | 41.6% |
|  | Above 5 years | Count | 3 | 49 | 52 |
|  |  | % within | 5.8% | 94.2% | 100.0% |
|  |  | % within Multiple Alleles | 100.0% | 57.0% | 58.4% |
|  |  | % of Total | 3.4% | 55.1% | 58.4% |
| Total | | Count | 3 | 86 | 89 |
|  |  | % within | 3.4% | 96.6% | 100.0% |
|  |  | % within Multiple Alleles | 100.0% | 100.0% | 100.0% |
|  |  | % of Total | 3.4% | 96.6% | 100.0% |
|  | | *P* value | 0.263 | | |

Significant level values set at p<0.05 were generated using Chi Square test.
