# Supplementary material for: Genetic diversity of Plasmodium falciparum parasite by microsatellite markers after scale-up of insecticide-treated bed nets in western Kenya
Source: Malar J. 2015 Dec 9;14:495. doi: 10.1186/s12936-015-1003-x (PMC4675068; doi:10.1186/s12936-015-1003-x)
Supplement: Supplementary file 2 — 10.1186/s12936-015-1003-x Unbiased expected heterozygosity for three study areas by age. [file 12936_2015_1003_MOESM2_ESM.docx]

|  | *H_e_* 2007 by Age | | | | | |
| --- | --- | --- | --- | --- | --- | --- |
|  | Asembo |  | Gem |  | Karemo |  |
| Locus | Under 5 yrs | Over 5 yrs | Under 5 yrs | Over 5 yrs | Under 5 yrs | Over 5 yrs |
| Polya | 0.9190 | 0.9214 | 0.9270 | 0.8673 | 0.8709 | 0.8778 |
| Pfg377 | 0.3632 | 0.5101 | 0.3000 | 0.5263 | 0.2973 | 0.3597 |
| PfPK2 | 0.8952 | 0.8367 | 0.8454 | 0.8187 | 0.8206 | 0.8506 |
| ADL | 0.9085 | 0.8750 | 0.9055 | 0.9175 | 0.9279 | 0.8971 |
| EBP | 0.8421 | 0.8488 | 0.9095 | 0.8575 | 0.8603 | 0.8163 |
| P195 | 0.6608 | 0.7914 | 0.6970 | 0.5952 | 0.7413 | 0.7518 |
| TAA60 | 0.8524 | 0.8258 | 0.8381 | 0.7624 | 0.7763 | 0.7900 |
| TAA109 | 0.8053 | 0.8258 | 0.8645 | 0.8116 | 0.8561 | 0.8197 |
| **Overall** | **0.7808** | **0.8044** | **0.7859** | **0.7696** | **0.7688** | **0.7704** |

Additional file 2: Table S2 Unbiased Expected Heterozygosity for Three Study Areas by Age

Unbiased expected heterozygosity (*H_e_*) in children below and over 5 years of age in Asembo, Gem and Karemo in 2007. The overall *H_e_* is shown in bold.
