# Supplementary material for: Genetic diversity of Plasmodium falciparum parasite by microsatellite markers after scale-up of insecticide-treated bed nets in western Kenya
Source: Malar J. 2015 Dec 9;14:495. doi: 10.1186/s12936-015-1003-x (PMC4675068; doi:10.1186/s12936-015-1003-x)
Supplement: Supplementary file 3 — 10.1186/s12936-015-1003-x Allele sizes and compositions (base pairs, X-axis) and frequency distribution (Y-axis) of eight individual MS for P. falciparum parasite populations from Asembo, Gem and Karemo areas, 2007. [file 12936_2015_1003_MOESM3_ESM.docx]

Additional file 3

Figure S1. Allele sizes and compositions (base pairs, X-axis) and frequency distribution (Y-axis) of eight individual MS for *P. falciparum* parasite populations from Asembo, Gem and Karemo areas, 2007. Standardized Y-axis scale (0-1) was used to depict the proportion of different alleles in each locus.
