# Supplementary material for: Genetic diversity of Plasmodium falciparum parasite by microsatellite markers after scale-up of insecticide-treated bed nets in western Kenya
Source: Malar J. 2015 Dec 9;14:495. doi: 10.1186/s12936-015-1003-x (PMC4675068; doi:10.1186/s12936-015-1003-x)
Supplement: Supplementary file 4 — 10.1186/s12936-015-1003-x Comparison of pairwise LD for P. falciparum populations in a) Aembo, Gem, and Karemo Areas, 2007 survey and, b) Asembo 1996, 2001 and 2007 surveys. [file 12936_2015_1003_MOESM4_ESM.docx]

Additional file 4: Table S3 Comparison of Pairwise Linkage Disequilibrium (LD) for *P. falciparum* Populations in a) Asembo, Gem and Karemo areas, 2007 Survey and, b) Asembo 1996, 2001 and 2007 Surveys

| 3a) | Pair-wise *p*-values of LD (p < 0.0018) in the Asembo, Gem and Karemo *P. falciparum* populations for the 2007 survey | | | | | | | | |
| --- | --- | --- | --- | --- | --- | --- | --- | --- | --- |
| Locus | Area | Alleles | Pfg377 | PfPK2 | ADL | EBP | P195 | TAA60 | TAA109 |
| Poly-α | Asembo | 16 | 0.0470 | **0.0001** | **0.0002** | 0.0053 | **0.0001** | **0.0000** | 0.0064 |
|  | Gem | 17 | 0.4226 | 0.0053 | **0.0000** | **0.0020** | **0.0009** | **0.0002** | 0.0138 |
|  | Karemo | 19 | 0.3193 | 0.0183 | 0.0608 | 0.1333 | **0.0002** | **0.0000** | 0.2703 |
| Pfg377 | Asembo | 5 |  | 0.0110 | 0.0513 | 0.0060 | 0.0039 | 0.0037 | 0.6002 |
|  | Gem | 6 |  | 0.0057 | **0.0000** | 0.2167 | 0.0131 | 0.3792 | 0.0140 |
|  | Karemo | 5 |  | 0.0356 | 0.1863 | 0.0053 | **0.0008** | 0.0401 | 0.1073 |
| PfPK2 | Asembo | 10 |  |  | **0.0000** | **0.0000** | **0.0000** | **0.0000** | **0.0001** |
|  | Gem | 11 |  |  | **0.0000** | **0.0004** | 0.0912 | **0.0004** | **0.0006** |
|  | Karemo | 12 |  |  | **0.0004** | **0.0001** | **0.0006** | 0.0023 | **0.0006** |
| ADL | Asembo | 12 |  |  |  | **0.0000** | **0.0000** | **0.0000** | 0.0068 |
|  | Gem | 14 |  |  |  | **0.0001** | **0.0012** | 0.0060 | **0.0000** |
|  | Karemo | 16 |  |  |  | **0.0001** | **0.0000** | **0.0000** | 0.0374 |
| EBP | Asembo | 8 |  |  |  |  | **0.0006** | **0.0010** | **0.0002** |
|  | Gem | 14 |  |  |  |  | **0.0000** | 0.0299 | 0.0086 |
|  | Karemo | 11 |  |  |  |  | 0.0040 | **0.0000** | 0.0283 |
| P195 | Asembo | 7 |  |  |  |  |  | **0.0002** | 0.0518 |
|  | Gem | 6 |  |  |  |  |  | 0.0139 | **0.0000** |
|  | Karemo | 6 |  |  |  |  |  | **0.0000** | **0.0002** |
| TAA60 | Asembo | 8 |  |  |  |  |  |  | 0.0121 |
|  | Gem | 9 |  |  |  |  |  |  | 0.0034 |
|  | Karemo | 7 |  |  |  |  |  |  | **0.0000** |
| TAA109 | Asembo | 9 |  |  |  |  |  |  |  |
|  | Gem | 12 |  |  |  |  |  |  |  |
|  | Karemo | 14 |  |  |  |  |  |  |  |

The number of gene copies and alleles used to generate pairwise LD are shown in Additional file 5 Table 4.

| 3b) Pair-wise *p*-values of LD (p < 0.0018) of the *P. falciparum* parasite populations for Asembo 1996, 2001 and 2007 surveys | | | | | | | | |
| --- | --- | --- | --- | --- | --- | --- | --- | --- |
| **Locus** | **Survey** | Pfg377 | PfPK2 | ADL | EBP | P195 | TAA60 | TAA109 |
| Poly-α | 1996 | 0.0568 | 0.0037 | 0.0287 | 0.1107 | **0.0001** | **0.0002** | 0.1151 |
|  | 2001 | 0.0050 | **0.0004** | 0.0048 | 0.0089 | **0.0006** | 0.1143 | 0.0310 |
|  | 2007 | 0.0470 | **0.0001** | **0.0002** | 0.0053 | **0.0001** | **0.0001** | 0.0064 |
| Pfg377 | 1996 |  | 0.3421 | **0.0004** | 0.0433 | **0.0001** | 0.4687 | 0.0321 |
|  | 2001 |  | 0.0546 | 0.0032 | **0.0005** | **0.0007** | 0.0157 | 0.0232 |
|  | 2007 |  | 0.0110 | 0.0513 | 0.0060 | 0.0039 | 0.0037 | 0.6002 |
| PfPK2 | 1996 |  |  | **0.0013** | 0.0043 | **0.0001** | **0.0001** | 0.005 |
|  | 2001 |  |  | 0.0471 | 0.1925 | 0.0167 | 0.0143 | 0.0658 |
|  | 2007 |  |  | **0.0001** | **0.0001** | **0.0001** | **0.0001** | **0.0001** |
| ADL | 1996 |  |  |  | 0.038 | 0.0069 | **0.0002** | **0.0001** |
|  | 2001 |  |  |  | 0.1811 | 0.0867 | 0.0026 | 0.0281 |
|  | 2007 |  |  |  | **0.0001** | **0.0000** | **0.0001** | 0.0068 |
| EBP | 1996 |  |  |  |  | **0.0001** | 0.0015 | 0.0095 |
|  | 2001 |  |  |  |  | **0.0001** | 0.0573 | 0.1548 |
|  | 2007 |  |  |  |  | **0.0006** | **0.0010** | **0.0002** |
| P195 | 1996 |  |  |  |  |  | **0.0001** | **0.0001** |
|  | 2001 |  |  |  |  |  | 0.0563 | 0.0256 |
|  | 2007 |  |  |  |  |  | **0.0002** | 0.0518 |
| TAA60 | 1996 |  |  |  |  |  |  | **0.0001** |
|  | 2001 |  |  |  |  |  |  | 0.0102 |
|  | 2007 |  |  |  |  |  |  | 0.0121 |
