# Supplementary material for: Genetic diversity of Plasmodium falciparum parasite by microsatellite markers after scale-up of insecticide-treated bed nets in western Kenya
Source: Malar J. 2015 Dec 9;14:495. doi: 10.1186/s12936-015-1003-x (PMC4675068; doi:10.1186/s12936-015-1003-x)
Supplement: Supplementary file 5 — 10.1186/s12936-015-1003-x Number of gene copies and number of alleles per locus used in LD analyses for Asembo, Gem and Karemo. [file 12936_2015_1003_MOESM5_ESM.docx]

| **Number of gene copies and alleles used in LD analyses for three areas** | | | | | | |
| --- | --- | --- | --- | --- | --- | --- |
|  | Asembo | | Gem | | Karemo | |
| Locus | Number of gene copies | Number of Alleles | Number of gene copies | Number of Alleles | Number of gene copies | Number of Alleles |
| Polya | 112 | 16 | 174 | 17 | 184 | 19 |
| Pfg377 | 110 | 5 | 148 | 6 | 184 | 5 |
| PfPK2 | 106 | 10 | 166 | 11 | 178 | 12 |
| ADL | 96 | 12 | 154 | 14 | 180 | 16 |
| EBP | 100 | 8 | 168 | 14 | 174 | 11 |
| P195 | 106 | 7 | 164 | 6 | 174 | 6 |
| TAA60 | 112 | 8 | 172 | 9 | 174 | 7 |
| TAA109 | 106 | 9 | 164 | 12 | 168 | 14 |

Additional file 5: Table S4 Number of Gene Copies and Number of Alleles per Locus Used in the Linkage Disequilibrium (LD) Analyses for Asembo, Gem and Karemo
